# Pre-operative radiographic findings predicting concomitant posterior malleolar fractures in tibial shaft fractures: a comparative retrospective study

**DOI:** 10.1186/s12891-018-1982-1

**Published:** 2018-03-20

**Authors:** Zhipeng Huang, Yuan Liu, Wenjun Xie, Xiang Li, Xiaodong Qin, Jun Hu

**Affiliations:** 1 Department of Orthopedics, The First Affiliated Hospital of Nanjing Medical University, Nanjing, 210029 China; 2 Department of Infectious Diseases, The First Affiliated Hospital of Nanjing Medical University, Nanjing, 210029 China

**Keywords:** Tibial shaft fracture, Spiral, Posterior malleolar fracture, Radiograph

## Abstract

**Background:**

A concomitant tibial shaft and posterior malleolar fracture is a type of regular compound fracture. The associated posterior malleolar fractures are mostly occult fractures, which often do not show a fracture line on ordinary films, and thus lead to a high rate of misdiagnosis. The aim of the present study was to investigate factors helpful for the pre-operative detection of concomitant posterior ankle fractures using the ipsilateral radiographic tibia and fibula shaft fracture characteristics.

**Methods:**

One hundred eleven adult patients with tibial shaft fractures were selected using inclusion and exclusion criteria. Pre-operative ankle radiographs and computed tomography (CT) scans were obtained for all patients, and clinical data, including age and gender, were collected. Patients were divided into two groups (posterior malleolar fracture and no posterior malleolar fracture groups). Fracture height, fracture length, fracture shape, and Haraguchi type of posterior malleolar fracture were measured on radiographs and CT images, and were compared between the two groups. Multiple logistic regression analysis was performed to identify the factors that significantly contributed to concomitant posterior malleolar fractures. Receiver operating characteristic curves were calculated, and cut-off values were used to predict posterior malleolar fractures on pre-operative imaging measurements.

**Results:**

Of the 111 patients with tibial shaft fractures, 42 (37.8%) had a concurrent posterior malleolar fracture. Age, gender and affected side were not significantly different, but tibial fracture location, fracture length, and fibular and tibial fracture shape were significantly different between the two groups. In the multiple logistic analysis, tibial fracture location, fracture length, and tibial fracture shape were shown to be significant factors contributing to posterior malleolar fractures. Receiver operating characteristic curves showed that the status of tibial shaft fractures is closely related to the associated posterior malleolar fracture.

**Conclusion:**

Ipsilateral posterior ankle fractures are commonly associated with tibial shaft fractures, especially spiral-type injuries. An analysis of the imaging features of such fractures and evaluation of the diagnostic value of various methods can provide imaging basics for the development of accurate and appropriate treatment options.

## Background

Posterior malleolus fractures are a common type of ankle fracture with variations ranging from small posterior malleolus avulsion fractures to large displaced fractures. The integrity of the posterior malleolus and the posterior tibiofibular ligaments play an important role in load transfer of ankle joints, the stability of the back talus, and the stability of ankle joint rotation [1]. Posterior malleolus fractures account for 7%–44% of ankle fractures, and most of the posterior malleolus fractures occur in post-rotatory extorsion fractures [1]. Tibial shaft fractures with ankle fractures have recently attracted increasing attention in clinical practice. In this type of fracture, posterior malleolus fractures mostly manifest as crack fractures and non-obvious displacement, which are challenging to detect on X-ray films. Indeed, the misdiagnosis rate on x-ray films is 67.9% – 91.2% [2]. Therefore, how to optimize the imaging examination method to reduce the misdiagnosis rate is an important clinical problem.

The aim of this study was to determine factors helpful for the pre-operative detection of concomitant posterior ankle fractures using the ipsilateral radiographic tibia and fibula shaft fracture characteristics. It was hypothesized that the morphologic features of tibial shaft fractures contribute to posterior malleolus fractures.

## Methods

This study was approved by the clinical research ethics committee of the First Affiliated Hospital of Nanjing Medical University. All patients with tibial shaft fractures underwent CT examinations between 2012 and 2017; the obtained data was retrospectively analyzed. The exclusion criteria were as follows: fractures primarily involving the knee joint (tibial plateau fractures); fractures primarily involving the ankle joint (uni-, bi-, and trimalleolar ankle fractures); old fractures or fractures due to unhealed fractures; congenital dysplasia; neuromuscular disorders; infections; bone tumors and other diseases possibly altering the normal anatomy of skeletal muscle; bilateral comminuted tibial shaft fractures; and fractures without ipsilateral ankle CT images or fractures with X-ray filming excluding the full length of the tibia and fibula [3].

Demographic data, including age and gender, were recorded. As part of our institution’s standard pre-operative evaluation, all tibial shaft fracture patients have orthogonal tibia radiographs with supplemental radiographic views of the ankle and knee. A CT scan without iodinated contrast of the entire tibia that included the ankle was also obtained to assess for concomitant injuries. All CT scans were performed on a 64-slice scanner utilizing 1.25-mm axial slices (Siemens SOMATOM Definition AS 64-slice spiral CT machine [detector dimension, 64 * 1.25 mm, pitch, 0.8, tube voltage, 130 kV, 80 ~ 520 mAs]; Shanghai, China).

Pre-operative radiographs were independently reviewed by three orthopedic surgeons. X-ray films were preliminarily analyzed according to the location of the tibia and fibula fractures. Tibial shaft fractures were divided into four types (transverse, oblique, spiral, and complex). Fibular and tibial fracture location was defined as the midpoint of the fracture line, and the point was calculated as the percentage between the ankle and knee joint lines, where the ankle joint line was 0% and the knee joint line was 100%. Similarly, fibular fracture location was calculated as the percentage between the distal and proximal tips of the fibula. Fracture length was defined as the vertical height between the lowest and highest points of the fracture line of the tibial shaft [3].

Three-dimensional evaluation of posterior malleolus fractures with tibial shaft fractures was performed in cross-sectional, sagittal, and coronal surfaces. If the posterior malleolus fracture involves > 25% of the articular surface or fracture displacement is > 2 mm, fixation of the posterior ankle injury (distal tibial articular surface) by surgery is necessary [4].

A technique of mapping fracture lines for a given bone, which has been previously described, was used for this study [5–7]. A brief description follows. The CT axial cut located 3 mm above the distal tibial subchondral surface from each of the 42 patients was selected for analysis and fracture “mapping.” Each image was obtained digitally, and subsequently enlarged to fit a grid to standardize the size of the images, which were then uploaded into a graphics design program (Macromedia Fireworks MX software; Macromedia, Inc., San Francisco, CA, USA). To identify the common fracture patterns, all 42 images were divided into 2 sides and were superimposed to create a “heat map” of the plafond. These superimposed images resulted in a frequency diagram based on the density of the fracture lines.

The inter-observer reliability of the three orthopedic surgeons was evaluated using intra-class correlation coefficients (ICCs). The surgeons measured the radiologic indices independently without having access to the patients’ clinical information and the measurement results of other surgeons. One of the orthopedic surgeons repeated the measurements of the radiologic indices 3 weeks after the observer reliability test to assess the reliability of the intra-observer. Inter- and intra-observer reliability testing was performed on 111 patients. After the reliability test, all flat sheet measurements were done by one of the authors who performed intra-observer reliability testing.

Descriptive statistics, including the mean, standard deviation, and proportions, were determined. A Kolmogorov-Smirnov test verified the normality of the distribution of continuous variables. Comparisons between the patients with and without concomitant posterior malleolar fractures were performed using a t-test or Mann-Whitney U test and a chi-square test according to the data characteristics. To combine the factors that significantly contributed to predict posterior ankle fractures, multiple logistic regression with the stepwise selection method was used. The sensitivity and specificity of the receiver operating characteristic curve were calculated for each radiographic measurement. All statistics were two-tailed, and a *p* value < 0.05 was considered significant [8].

## Results

**Fig. 1:**
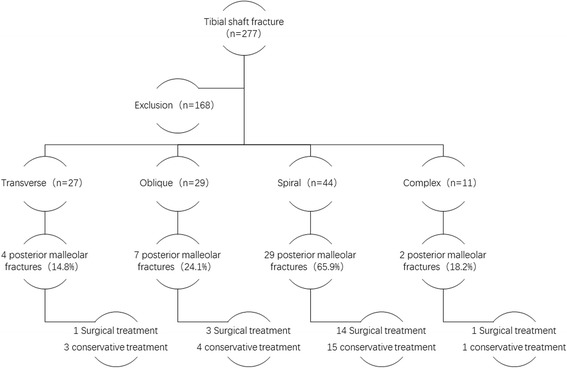
According to tibial fracture shape, our study consisted of 27 transverse, 29 oblique, 44 spiral, and 11 complex fractures

In this study, a total of 111 patients with tibial shaft fractures met the inclusion and exclusion criteria. Of these patients, 72 were males and 39 were females, with an average age of 46.6 ± 16.1 years. Of the 111 patients with tibial fractures, 42 (37.8%) had ipsilateral posterior ankle fractures. Of 42 ankle joint injuries, 30 were isolated posterior malleolus fractures, 11 were posterior malleolus fractures combined with lateral malleolus fractures, and 1 was a posterior malleolus fractures combined with lateral and medial malleolus fractures. In 42 patients with posterior malleolus fractures, 19 underwent surgical treatment and 23 underwent conservative treatment (Fig. 1).

**Fig. 2:**
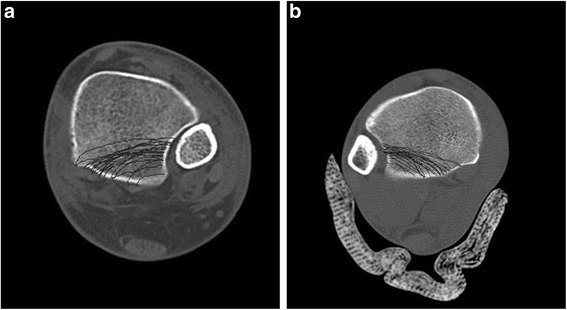
Axial CT (**a** right side, **b** left side) scans demonstrating the posterior malleolus fracture pattern. The Posterior Malleolar Map revealed a fracture pattern and morphology consistent with the fracture lines, as originally described by Haraguchi

After critically reviewing all of these images, consistent fracture lines were identified and deemed major fracture lines. We identified two major fracture lines, as follows: 1) larger posterolateral-oblique type fragments (Haraguchi type I); and 2) small posterolateral avulsion type fragments (Haraguchi type III; Fig. 2).

Radiographic measurements showed satisfactory inter- and intra-observer reliabilities (Table 1). A comparison between the groups with and without posterior malleolar fractures, univariate logistic regression analysis indicated that there were no significant differences in age, gender, mechanism of injury, fibular fracture location, and fibular fracture shape, but there were significant differences in the location of tibial fractures, fracture length, and tibial fracture shape (*p* < 0.05; Table 2).

**Table 1:** Intra- and inter-observer reliabilities of measurements

Measurements	Intra-observer Reliability		Inter-observer Reliability	
	ICC	95% CI	ICC	95% CI
Tibial fracture location, %	0.989	0.978 to 0.997	0.917	0.856 to 0.958
Fibular fracture location, %	0.949	0.876 to 0.981	0.926	0.836 to 0.961
Fracture length, %	0.891	0.808 to 0.949	0.808	0.701 to 0.912

*Abbreviations*: *ICC* Intraclass correlation coefficient, *CI* Confidence interval

**Table 2:** Comparison between groups with and without posterior malleolar fractures

	With posterior malleolar fracture	Without posterior malleolar fracture	*P* Value
Patients	42	69	
Age, y^a^	46.79 ± 14.06	46.49 ± 17.33	0.93
Gender			0.92
Male	27	45	
Female	15	24	
Side			0.96
Right	19	31	
Left	23	37	
Bilateral	0	1	
Fracture location, %^a^			
Tibial	27.52 ± 10.08	37.59 ± 18.08	< 0.01
Fibular	38.94 ± 34.53	38.68 ± 27.94	0.97
Fracture length, %^a^	15.11 ± 6.08	11.31 ± 9.52	< 0.05
Fibular fracture shape			< 0.01
Intact	5	10	
Proximal level	13	11	
Middle level	2	24	
Distal level	20	21	
Complex	2	3	
Tibial fracture shape			< 0.01
Transverse	4	23	
Oblique	7	22	
Spiral	29	15	
Complex	2	9	
Haraguchi Type			
Type I	41		
Type II	0		
Type III	1		

^a^The values are given as the mean and the standard deviation

We then further analyzed age, gender, mechanism of injury, tibial and fibular fracture location, fracture length, and tibial and fibular fracture shape using multiple logistic regression model, and the results showed that tibial fracture location, fracture length, and tibial fracture shape were significant contributing factors to posterior malleolar fracture injuries in tibial fractures (Table 3).

**Table 3:** Logistic regression analysis (Stepwise selection method)

Variable	Estimate	Standard Error	Odds Ratio	95% CI	*P* Value
Tibial fracture location, %	−0.05	0.02	0.96	0.93 to 0.99	< 0.01
Fracture length, %	0.05	0.03	1.05	1.00 to 1.11	< 0.05
Tibial fracture shape					
Spiral	2.32	0.70	10.22	2.57 to 40.56	< 0.01

**Fig. 3:**
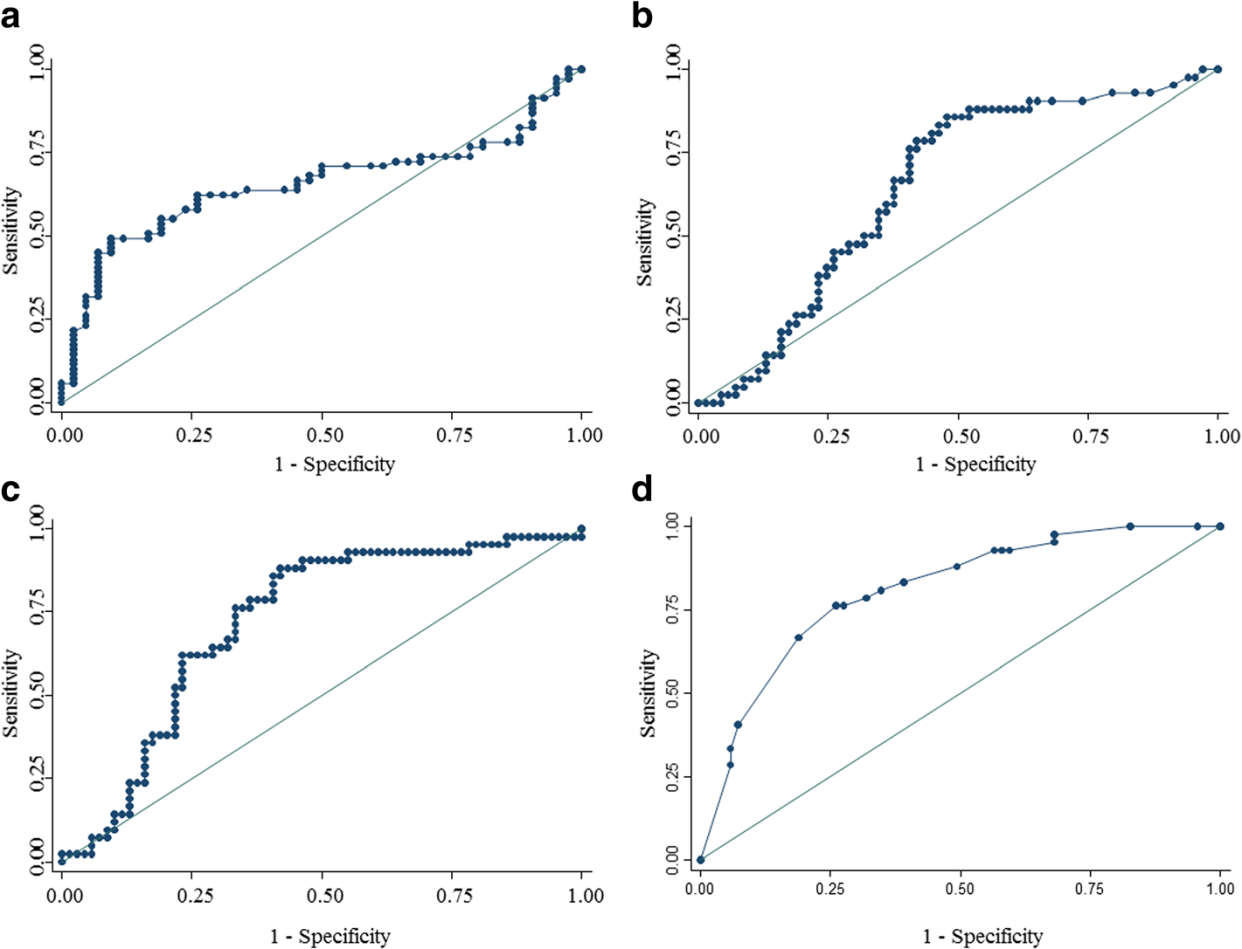
The receiver operating characteristic curve of the pre-operative radiographic measurements on the anteroposterior tibia and fibula view between the with posterior malleolar fracture group and without posterior malleolar fracture group. **a** the tibial fracture location; (**b**) fracture length; (**c**) combination between tibial fracture location and fracture length; (**d**) combination between shape of the tibial shaft fractures and fibular shaft fractures

The receiver operating characteristic curves for tibial fracture location and fracture length on radiographs of posterior ankle fractures were also calculated. The cut-off value for tibial fracture location was 40.0%, with a sensitivity of 92.9% and a specificity of 44.9% (Fig. 3a). The cut-off value for fracture length was 11.4%, with a sensitivity of 85.7% and a specificity of 52.2% (Fig. 3b). In addition, we analyzed the combination between tibial fracture location and fracture length. And we analyzed the combination between the shape of the tibial shaft fractures (transverse, oblique, spiral, and complex) and the shape of fibular fractures (intact, proximal, middle, distal, and complex). We found that the area under the curve for the combination of tibial fracture location and fracture length was 0.7208, with a sensitivity of 40.5% and a specificity of 78.3% (Fig. 3c). The area under the curve for the combination of tibial and fibula fracture shapes was 0.8094, with a sensitivity of 66.7% and a specificity of 81.2% (Fig. 3d).

## Discussion

The present study investigated the factors that could be used to detect posterior malleolar fractures concurrent with tibial shaft fractures pre-operatively and to provide cut-off values based on pre-operative radiographic measurements. Tibial fracture location, fracture length, and tibial fracture shape were significant pre-operative factors that were associated with posterior malleolar fractures. When the tibial fracture location is ≤40% or the fracture length is > 11.4% on a radiograph, posterior malleolar fractures should be suspected, appropriately evaluated, and managed at the time of tibial shaft fracture surgery.

The incidence of tibial shaft fractures combined with posterior malleolus fractures in each region show remarkable differences. Kempegowda [9] identified 1113 cases of tibial shaft fractures, 96 of which were associated with ankle fractures, thus accounting for 8.6% of all cases. Schottel and others [10] studied 71 cases of tibial shaft fractures, 35 of which showed combined ankle fractures, thus accounting for 49.3% of all cases. Stuermer et al. [11] reported that 43 (20.1%) of 214 patients with a tibial fracture were found to have an associated injury of the ankle joint. Hou Z et al. [12] found that 288 cases of tibial shaft fractures, 28 of which showed combined posterior malleolar fractures, thus accounting for 9.7% of all cases. Based on the previous studies, the incidence of tibial shaft fractures and concomitant ankle injuries had relatively large differences. We showed that the incidence of tibial shaft fractures combined with posterior malleolus fractures was 37.8% in our study.

The misdiagnosis of tibial shaft fractures combined with ankle fractures reflects the methods of examination. The lack of understanding of this concomitant injury is also an important reason resulting in misdiagnosis. At the time of initial consultation, most orthopedic surgeons only noticed a significant shift in the tibial shaft fracture, but often did not take into account the possible concomitant ankle fracture. When X-ray films were obtained, the ankle joint was not included, and the ankle fracture was therefore missed. Even if most X-ray examinations include the distal tibia, shooting angle and clarity also largely affect the diagnosis of fractures. It is challenging to visualize tibial ankle fractures from frontal films, and it is even more challenging to view the fractures from lateral radiographs due to the overlap with the fibula, especially when the fracture is combined with distal fibula fractures. In addition, most ankle fractures of this type are occult fractures, and it is difficult to diagnose these fractures through X-ray examinations. In these cases, non-displaced distal ankle fractures are often misdiagnosed [10].

Tibial shaft fractures combined with posterior malleolus fractures are a regular combined injury, and the cause of injury, mechanism of injury, and epidemiologic characteristics are different from ankle fractures. It has been shown that the incidence of posterior malleolus fractures in tibial shaft fractures combined with ankle joint injuries is significantly higher than in patients with ankle fractures only [13] .Ankle fractures are most commonly found in Lauge-Hansen classification degree III or above post-rotatory extorsion fractures and degree IV pronation-external rotation fractures. The typical posterior ankle coronal fractures often occur in rotational injuries. When the foot is in the pronation or posterior rotation position, the lower tibiofibular ligament injury may be injured upon external force on the talus due to rotation or valgus. One side of this ligament is attached to the distal posterior margin of the tibia, which is the posterior ankle. Posterior malleolus fractures are caused by avulsion of the lower tibiofibular ligament. At present, the mechanism of tibial shaft fractures combined with posterior malleolus injuries is still controversial. Some researchers believe that the fracture is mostly caused by indirect low-energy torsional forces. The mechanism of injury is due to rotation to the outside during forward movement because of inertia when the ankle is fixed, resulting in a spiral fracture in the weak parts involving one-third of the distal tibia. The fracture line goes from the inside lower aspect to the outside upper aspect, the ankle fracture is due to the talus shear during the movement upon sudden fixation of the foot or due to avulsion fractures caused by traction between the ankle and lower tibiofibular posterior ligament [14] .There are still some other types of tibial shaft fractures that cannot be included in the situation we discussed herein. Some scholars have stated that when tibial shaft fractures occur, the lateral leg muscles contract, resulting in excessive plantar flexion of the ankle joint, extrusion of the tibial fossa trailing edge by the talus, and thus ankle fractures [15]. Nevertheless, the mechanism leading to damage needs to be verified by further biomechanical studies.

Among the 42 posterior malleolus fractures in this study, 19 patients underwent surgical treatment and 23 patients were conservatively treated. Surgical fixation is necessary if the posterior ankle fracture is > 25% of the tibial articular surface or the fracture displacement is > 2 mm [4]. Fitzpatrick et al. [16] found that the mean and peak contact stress did not increase significantly, even in the cases in which posterior malleolar fracture involve > 50% of the articular surface. The results of the Fitzpatrick et al. [16] study showed that in a posterior malleolus fracture model, contact stress of the ankle joint is redistributed to the anterior medial side during movement of the ankle joint. This stress concentration may lead to changes in the weight load of the cartilage within the contact interface, resulting in the final induction of post-traumatic arthritis. Drijfhout van Hooff et al. [17] showed that the fixation should be implemented when the posterior malleolus fracture block is > 5%; an articular surface step > 1 mm will lead to the development of osteoarthritis.

The limitations of the current study include those inherent to the retrospective design. Secondly, intra-observer reliability is better to be calculated from the data of the 3 orthopedics. In addition, in our study all tibia shaft fracture surgery was used an intramedullary nail fixation, but because posterior malleolar fractures were performed by different surgeons and different surgical approaches were adopted, including hollow screws and buttress plates, we cannot discuss the choice of surgical methods, and we cannot evaluate functional outcomes after fixation of posterior malleolus fractures with a tibial shaft fracture. Boraiah et al. [18] reported on a series of 24 patients of posterior malleolar fractures associated with tibia fractures, where the malleolus was fixed before intramedullary nailing of the tibia, and there were no complications during the post-operative follow-up. Kempegowda et al. [9] strongly suggests that malleolar fractures should be fixed before nailing of the tibia to reduce the risk of intra-operative displacement and poor reduction. The Kempegowda et al. [9] study was based on a small number of patients, bringing into question the power of the results. Sufficiently powered, good-quality, randomized trials are warranted.

## Conclusions

Our clinical experience shows that it is necessary to have pre-operative regular radiography, including frontal and lateral X-ray films of ankle joints and carefully check the films, which are critical the diagnosis. For the suspected cases, it is important to perform a CT examination to determine whether or not an ankle fracture is present. In the current study, we performed CT examinations for all diagnosed and suspected tibial shaft fractures with ankle fractures. For the regions that lack medical resources or with high medical costs, diagnosis according to the pre-operative films can save a substantial amount of medical costs. For combined ankle fractures with tibial shaft fractures in busy trauma centers, if ipsilateral ankle joint CT films are not obtained, surgeons can also determine concomitant ankle fracture according to analysis of characteristics in flat films, which can help intra-operative restoration and avoid misdiagnosis.

## Abbreviations

CT: Computed tomography
ICCs: Intra-class correlation coefficients
